# Aligning Dietary Intake and Food Purchases in U.S. Older Adults: Insights from NHANES and Circana Consumer Network

**DOI:** 10.1093/geroni/igaf122.2039

**Published:** 2025-12-31

**Authors:** Naglaa El-Abbadi, Marcus Lehr, Frederick Nusetor, Cristina Moraga Franco, Andrea Carlson, Kyla Shea, Chao-Qiang Lai, Joshua Rutt

**Affiliations:** Jean Mayer USDA Human Nutrition Research Center on Aging, Boston, Massachusetts, United States; Jean Mayer USDA Human Nutrition Research Center on Aging, Boston, Massachusetts, United States; Jean Mayer USDA Human Nutrition Research Center on Aging, Boston, Massachusetts, United States; Jean Mayer USDA Human Nutrition Research Center on Aging, Boston, Massachusetts, United States; U.S. Department of Agriculture, Washington, District of Columbia, United States; Tufts University, Boston, Massachusetts, United States; USDA ARS, Human Nutrition Research Center on Aging at Tufts University, Boston, Massachusetts, United States; Jean Mayer USDA Human Nutrition Research Center on Aging, Boston, Massachusetts, United States

## Abstract

Diet quality is critical for healthy aging, yet assessing dietary intake remains complex. Nationally representative datasets provide valuable but distinct perspectives. The National Health and Nutrition Examination Survey (NHANES) captures self-reported consumption, while the Circana Consumer Network (Circana) records grocery purchases of approximately 60,000 households. The objective of this analysis was to explore whether dietary patterns observed in NHANES 2017-2018 align with food purchase trends in Circana 2018 in U.S. adults 55 years and older. Dietary intake data were derived from single 24-hour recalls (NHANES n = 2132), or grocery food purchase from 1- and 2-person households (Circana n = 26432). We also conducted stratified analyses by sex and age group (55-64, 65-74, 75+). Diet quality was scored using the 2020 Healthy Eating Index (HEI), and food group composition was evaluated by % weight (g) and energy (kcal). The mean±SE total HEI in NHANES was 52.1±0.9. Though overall HEI scores improved with age in both datasets, neither dataset suggests that older adults consistently meet dietary recommendations. Older adults were below HEI component adequacy/moderation recommendations for Fruit, Greens/Beans, Whole Grains, and Saturated Fats; these also showed greatest variation by age. While food group proportion by mass aligned between consumption and purchase for various food groups, there were key disparities in Dairy, Mixed Dishes, Condiments, and Snacks/Sweets; and Vegetables by caloric energy. These observations underscore the potential for integrating food purchase data with dietary intake assessments to enhance understanding of older adults’ dietary behaviors. Further research should explore demographic and socioeconomic factors influencing these patterns.

